# Perspectives on engineering and biomaterials in tampon design: a review of device-tissue interactions and dysmenorrhea risk

**DOI:** 10.3389/fbioe.2026.1920190

**Published:** 2026-09-16

**Authors:** Neriah Otu, Moriah Inyang-Otu

**Affiliations:** Schulich School of Engineering, University of Calgary, Calgary, AB, Canada

**Keywords:** biomaterials, biomedical engineering, chemical exposure, dysmenorrhea, intravaginal device, menstrual pain, tampon, vaginal physiology

## Abstract

**Background:**

Dysmenorrhea affects approximately 71% of menstruating individuals globally and is associated with substantial losses in quality of life, work and school participation, and healthcare utilization. Tampons are among the most widely used intravaginal menstrual devices, yet their design has evolved primarily around absorbency, structural integrity, and infection risk, not around comfort or pain minimization as a formal design or regulatory endpoint. No existing review has examined tampon design, materials, or biomechanical properties through the lens of menstrual pain or discomfort.

**Objectives:**

This review (1) synthesizes evidence on tampon-associated discomfort; (2) identifies plausible biomechanical and biological mechanisms linking device properties to pain; (3) characterizes tampon materials and biocompatibility; and (4) proposes an engineering framework for developing lower-discomfort products.

**Methods:**

Literature was searched across PubMed, Google Scholar, and regulatory databases. A PRISMA-informed screening process prioritized primary research and systematic reviews, identifying a core evidence base of 51 sources for narrative synthesis.

**Results:**

No randomized controlled trials or prospective clinical studies directly examining the effect of tampon use or tampon material type on menstrual cramp severity were identified. However, evidence from adjacent fields supports several plausible mechanisms: colposcopy studies of older superabsorbent tampon formulations documented mucosal desiccation and microulceration, though modern products have not been comparably characterized; tampon materials contain detectable chemical residues including metals, phthalates, and volatile organic compounds, though their bioavailability across vaginal mucosa and contribution to systemic exposure remain unquantified, and current regulatory reviews (FDA, American College of Medical Toxicology) have not identified an associated safety concern; tampon use may perturb the vaginal microbiome in ways that could increase pro-inflammatory bacterial activity; and removal dynamics plausibly generate friction and contact pressure against vaginal tissue, though this has not been directly measured. These mechanisms are mechanistically coherent hypotheses that may converge in populations with elevated pain sensitivity, not established causal pathways.

**Conclusion:**

A substantial evidence gap exists at the intersection of tampon design and menstrual pain. Biomedical engineering provides a pathway to close this gap *via* testable design specifications. These include friction-reducing surface architectures and controlled expansion geometry, which are framed as research targets requiring further validation.

## 1 Introduction

Dysmenorrhea (defined as uterine cramping pain occurring in association with menstruation) is among the most prevalent and undertreated conditions affecting reproductive-age individuals worldwide (De Arruda et al., 2026). A 2026 systematic review and meta-analysis spanning 336 studies across 70 countries estimated the pooled global prevalence of dysmenorrhea at 71.3% (95% CI 68.7%–73.8%), with primary dysmenorrhea alone affecting approximately 73% of those who menstruate (De Arruda et al., 2026). In Canada, a national survey of menstruating women found that approximately 60% met clinical criteria for primary dysmenorrhea, with more than half reporting their pain as moderate or severe (Burnett et al., 2005). Despite this prevalence, dysmenorrhea remains chronically underresearched relative to its burden, a gap reflected in the absence of any direct clinical trials examining tampon-associated pain–the central motivation for this review.

The consequences of undertreated menstrual pain extend well beyond individual suffering. Endometriosis, a condition defined in part by severe dysmenorrhea, affected an estimated 22.28 million people globally in 2021 according to the Global Burden of Disease study (Yu et al., 2025), and more than two-thirds of those affected have reported missing school or work due to pain (Yu et al., 2025; Serya et al., 2025). The economic cost of endometriosis-related absenteeism and reduced workplace productivity in the United States has been estimated at approximately USD 45.4 billion annually (Soliman et al., 2017). It should be noted that this study was funded by AbbVie Inc., a manufacturer of endometriosis treatment medication. More broadly, dysmenorrhea across all causes contributes to a mean 1.3 days of work absenteeism and 8.9 days of reduced-productivity presenteeism per person per year in large population-based cohorts (Serya et al., 2025; Nariai et al., 2025). These figures understate the true burden among individuals who continue to attend school or work while in pain (Sasha et al., 2023).

Tampons are one of the most widely used intravaginal menstrual products, particularly in North America and Europe. The global tampon market was valued at approximately USD 6.5 billion in 2025, with projected continued growth ^1^. In the United States, approximately 50% of non-Hispanic White menstruators report tampon use as their primary product, though tampon uptake is substantially lower among Black, Hispanic, and Asian menstruators, and among individuals of lower socioeconomic status (Zota et al., 2023; Wang et al., 2025). In Canada, period poverty is a significant public-health concern that disproportionately affects marginalized and lower socioeconomic-status groups (Lee et al., 2024).

Tampons are regulated as Class II medical devices in both the United States by the Food and Drug Administration (FDA) and in Canada by Health Canada (Health Canada, 2026; Pollard, 2023). Regulatory premarket review requirements focus primarily on absorbency standardization, structural integrity, and toxic shock syndrome (TSS) risk; they do not include pain or comfort as required endpoints. Product-level innovation has continued in the decades since: manufacturers have engineered fiber cross-sectional geometry to improve absorbency without reliance on the superabsorbent materials withdrawn after the 1980s toxic shock syndrome epidemic (Harter et al., 2024), and internal density gradients to reduce leakage (Hou and Acton, 2016). However, menstrual health historians have documented that testing standards and regulatory oversight have not kept pace with these innovations (Vostral et al., 2020; Reame et al., 2020), and pain or comfort has never been established as a required or independently validated design endpoint.

This review examines whether a case can be made, from evidence or from first principles, that tampon materials, geometry, and biomechanical properties could contribute to menstrual pain or pelvic discomfort in some users. It is not the premise of this review that tampons cause cramps. The primary dysmenorrhea mechanism is well established as prostaglandin-driven uterine smooth muscle hypercontractility (Barcikowska et al., 2020) independent of product use. However, a growing body of evidence supports the possibility that tampon material properties interact with vaginal physiology in ways that could aggravate pain in susceptible individuals through mucosal irritation, chemical exposure, microbiome disruption, or mechanical pressure. These interactions have not been systematically studied.

This review makes four contributions to the literature. First, it synthesizes and evaluates available evidence on tampon-associated discomfort and the current absence of direct pain outcome data. Second, it identifies plausible biological and biomechanical mechanisms that should inform future investigation. Third, it integrates a health equity lens, recognizing that the populations most burdened by menstrual pain are precisely those most underserved by current product design and research. Fourth, it proposes a biomedical engineering design framework for developing and evaluating a lower-discomfort tampon as a tractable translational target.

## 2 Search strategy

This narrative review was conducted using PubMed/NCBI, Google Scholar, ScienceDirect, Wiley Online Library, Frontiers, and regulatory databases including FDA.gov and Canada.ca/Health Canada. Preprint servers (bioRxiv, medRxiv) were searched for emerging evidence, with preprints noted and excluded from claims requiring established evidence. Government, regulatory, and parliamentary sources were included as grey literature only for regulatory, policy, surveillance, or public-health context. These sources were not used as primary scientific evidence for biological, clinical, or mechanistic claims.

Primary search terms included: tampon dysmenorrhea; tampon menstrual cramps; tampon pelvic pain; tampon discomfort; tampon vaginal pain; tampon materials cotton rayon; tampon absorbency toxic shock syndrome; tampon microbiome; menstrual product materials; menstrual product safety; organic cotton tampons; vaginal mucosa tampon; vaginal microbiome menstrual products; tampon chemical exposure; tampon volatile organic compounds; tampon heavy metals; tampon bleaching dioxins; menstrual product regulation Canada; menstrual pain racial disparities; dysmenorrhea Black women; endometriosis diagnostic delay Black women; fibroids Black women prevalence; menstrual equity Canada; biomaterials vaginal products; vaginal drug delivery biomaterials; biomechanics tampon design; tampon expansion pressure friction absorbency.

This review was informed by the criteria of the Scale for the Assessment of Narrative Review Articles (SANRA) and incorporated a PRISMA-informed record-flow diagram to transparently summarize the volume of literature identified and assessed (Figure 1) (Haddaway et al., 2022). A protocol was not registered in PROSPERO, and formal deduplication and dual-independent screening were not performed, consistent with the narrative design of the review. Accordingly, the methods and findings should not be interpreted as those of a systematic review.

**FIGURE 1:**
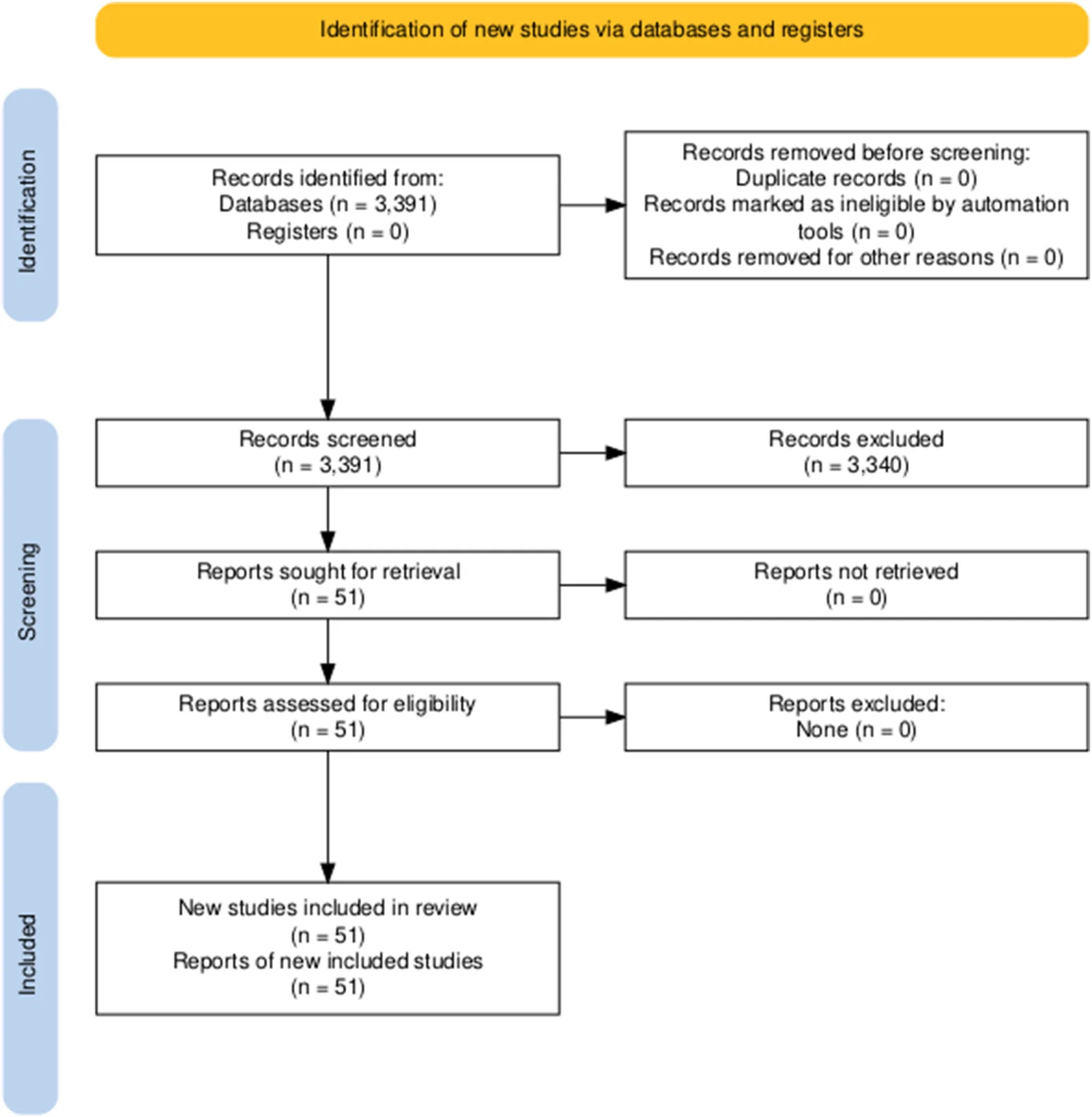
PRISMA-informed flow diagram of the literature identification and selection process. The diagram summarizes records identified through database searching, followed by screening, eligibility assessment, and inclusion in the narrative synthesis. Generated using the PRISMA2020 R package and Shiny app (Haddaway et al., 2022).

The 27 search strings described above were run in PubMed, producing an aggregate, non-deduplicated total of 3,391 results. This total represents the sum of the results returned by each search and includes substantial overlap among searches targeting related literature. Sources were assessed for relevance by the authors, with emphasis placed on primary research and recent systematic reviews, and the database search was supplemented by citation searching from key reviews. Together, these methods produced a core evidence base of 51 sources included in the narrative synthesis. Because supplementary citation searching was not separately tracked or quantified, it is not depicted as a distinct identification pathway in Figure 1.

The complete reference list contains 57 citations. The remaining six citations comprise three patents discussed in Section 12 (Harter et al., 2026; Glassman, 1991; Agyapong, 2010), two targeted sources added during revision to support mechanistic claims in Section 5.3 (Giovannetti et al., 2023; Arion et al., 2024), and one grey-literature or regulatory source used for contextual information rather than as primary evidence.

To examine the central finding that no clinical trial has directly evaluated tampon material or design in relation to menstrual pain, an additional targeted PubMed search was conducted. A search combining terms for tampons, menstrual pain, and clinical trials returned nine results, while a search combining tampon with the randomized controlled trial publication-type filter returned 92 results. Many of the latter concerned surgical applications or nasal packing rather than menstrual tampons. No study directly comparing tampon material or design in relation to dysmenorrhea or pelvic pain outcomes was identified, supporting the evidence gap described in this review.

Primary research studies were prioritized throughout. Systematic reviews and meta-analyses were included for epidemiological estimates and to identify primary studies. Narrative reviews were used for framing and mechanisms. Studies published within the last 5 years (2021–2026) were preferentially cited for primary data; foundational studies older than 5 years were retained where no recent equivalent existed, and this is noted explicitly.

Evidence quality is labeled throughout this review as: established (replicated, mechanism-supported), preliminary (limited primary data), indirect (adjacent evidence without direct tampon-pain studies), speculative (mechanistically plausible but not empirically tested), or based on user reports or passive surveillance (MAUDE data, adverse event reports).

## 3 Current menstrual product landscape

### 3.1 Product types and market context

Menstrual products include internally worn devices (tampons, menstrual cups, menstrual discs) and externally worn products (sanitary pads, panty liners, period underwear) (Van Eijk et al., 2019). Tampons consist of a compressed absorbent pledget (typically composed of cotton, rayon/viscose, or a blend of both) inserted into the vaginal canal to absorb menstrual fluid. They are available with or without an applicator, in graduated absorbency tiers (junior, regular, super, super plus), and are designed for single use.

Global tampon sales have been driven by factors including increasing female labor force participation, urbanization, and growing awareness of menstrual hygiene options in low- and middle-income countries^1^. However, tampon use is not universal. Pads remain the predominant product globally, particularly in Asia, sub-Saharan Africa, and among lower-income populations in North America (Zota et al., 2023; Wang et al., 2025).

### 3.2 Use patterns by sociodemographic group

A 2023 US cross-sectional study of 661 menstruating individuals found that White participants were significantly more likely to use unscented tampons and menstrual cups, while Black participants were more likely to use vaginal douches and intimate wipes, products associated with distinct chemical exposure profiles (Zota et al., 2023). These differences persisted after adjustment for education, suggesting a role for cultural practice, product marketing, access, and provider communication beyond socioeconomic status alone (Zota et al., 2023). A larger cross-sectional analysis using the Apple Women’s Health Study (n = 11,455) confirmed that non-Hispanic Black participants reported the lowest tampon use rates (31% vs. 50% among non-Hispanic White participants) and that lower socioeconomic status was independently associated with predominantly pad use (Wang et al., 2025). These differential use patterns have implications for comparative chemical exposure across menstrual product types and for generalizability of tampon research conducted predominantly in White, higher-income populations.

### 3.3 Regulatory frameworks

In Canada, menstrual tampons are regulated as Class II medical devices under the Food and Drugs Act. Health Canada requires manufacturers to obtain a medical device licence, demonstrate quality manufacturing standards, and submit to post-market surveillance requirements. Tampon packaging must include standardized absorbency labelling and information on TSS risk and symptoms (Health Canada, 2026). In the United States, the FDA classifies tampons as Class II medical devices subject to 510(k) premarket notification, requiring manufacturers to demonstrate substantial equivalence to a predicate device, and to performance standards (Pollard, 2023). A 2023 review of FDA regulatory history noted that while safety testing requirements encompass biocompatibility and TSS risk, there is no specific requirement for tampon manufacturers to assess pain or discomfort as clinical endpoints (Pollard, 2023).

Both regulatory systems have historically treated tampons as products with a well-established safety profile, and both have come under renewed scrutiny following recent studies identifying metals, phthalates, and other chemicals in commercially available products (Shearston et al., 2024; Marroquin et al., 2024).

## 4 Tampon use and menstrual pain: What does the evidence show?

### 4.1 The absence of direct clinical evidence

A thorough search of the primary literature identified no randomized controlled trials, prospective cohort studies, or crossover clinical studies specifically designed to examine whether tampon use increases menstrual cramp severity, dysmenorrhea scores, or pelvic pain compared to other menstrual management methods. This absence constitutes the central evidence gap motivating this review.

The only prospective randomized comparison of tampons with an alternative product is the FLOW (Finding Lasting Options for Women) trial, a multicentre randomized controlled trial (RCT) (n = 110) comparing tampons to menstrual cups over two menstrual cycles (Howard et al., 2011). The FLOW trial reported higher overall satisfaction with menstrual cups vs. tampons on a seven-point Likert scale (mean 5.4 vs. 5.0, P = 0.04), and initially higher vaginal discomfort with cups that declined with continued use. Menstrual pain was not measured as an outcome in this trial (Van Eijk et al., 2019; Howard et al., 2011). No equivalent trial comparing different tampon types (cotton vs. rayon, organic vs. conventional) on pain outcomes exists.

### 4.2 User-reported discomfort: passive surveillance data

In the absence of primary outcome data, adverse event reporting provides some signal, albeit with significant limitations. A 2023 comprehensive tampon safety assessment analyzed FDA MAUDE (Manufacturer and User Facility Device Experience) adverse event reports and identified the following as the five most commonly reported adverse events for tampons with applicators: complication of device insertion (50.6%), vulvovaginal pain (39.8%), vulvovaginal discomfort (28.8%), vulvovaginal injury (23.8%), and foreign body in the reproductive tract (21.9%) (Hochwalt et al., 2023). These figures must be interpreted with caution: MAUDE is a passive reporting system subject to significant underreporting bias, reporting does not establish causation, and the denominator of total tampon use cycles is unknown. Nevertheless, the pattern, with pain and discomfort ranking among the most common reported concerns, suggests that tampon-associated discomfort is not negligible in the user population.

Health Canada’s safety review of U by Kotex Sleek and Click tampons, initiated following an increase in Canadian adverse event reports, similarly documented a cluster of reports involving vaginal irritation, abdominal pain, fever, nausea, and headache following use, in addition to tampon fragmentation events requiring medical attention (Health Canada, 2025). While the regulatory trigger in this case was structural failure rather than pain *per se*, the report illustrates that intravaginal device design can generate clinically meaningful discomfort outcomes.

### 4.3 Insertion pain in populations with Underlying pelvic conditions

The most direct data on tampon-associated pain comes from populations with pre-existing pelvic conditions, though this evidence is suggestive rather than tampon-specific. A national incidence survey of U.S. women found that those with vulvodynia were 2.15 times more likely to report pain or discomfort with their first-time tampon use, and 2.4 times more likely to use a combined tampon-and-pad approach than women without vulvodynia (Marroquin et al., 2024). Because vulvodynia is defined by pain on vulvar contact generally, including intercourse and speculum examination, this finding likely reflects generalized touch-evoked hypersensitivity rather than a tampon-specific mechanism. It nonetheless demonstrates that intravaginal device insertion is a clinically meaningful pain trigger in a sensitized population, and that tampon insertion pain is prevalent enough in this group to function as a clinical screening indicator.

More broadly, conditions including vaginismus, pelvic floor hypertonicity, provoked vestibulodynia, endometriosis, adenomyosis, and uterine fibroids are associated with heightened pelvic mechanosensitivity and visceral hypersensitivity (Barcikowska et al., 2020; Reina et al., 2025; Serrano-Imedio et al., 2022). For individuals with these conditions, intravaginal device use, including tampons, may engage pain pathways that are not relevant in the general population. The current absence of condition-stratified tampon discomfort data represents a critical gap.

### 4.4 Summary

As established in Section 4.1, no direct clinical evidence exists linking tampon use to dysmenorrhea severity. Available evidence, from adverse event surveillance and subgroup studies in vulvodynia populations, suggests that tampon-associated pain and discomfort are clinically reported but have not been rigorously characterized. In populations with pre-existing pelvic pain conditions, tampon use is an identified discomfort trigger. Whether tampon material type or design features modify this risk is unknown.

## 5 Biological and physiological mechanisms relevant to tampon-associated discomfort

### 5.1 Dysmenorrhea: prostaglandins, uterine contractility, and central sensitization

Primary dysmenorrhea arises from progesterone withdrawal at the onset of menstruation, which drives upregulation of cyclooxygenase-2 (COX-2) in the uterine endometrium and a subsequent surge in prostaglandin synthesis (Barcikowska et al., 2020). Prostaglandin F2-alpha (PGF2α) induces intense uterine smooth muscle contraction and vasoconstriction, reducing uterine perfusion and producing ischemic pain, while prostaglandin E2 (PGE2) sensitizes afferent nerve endings to pain-producing stimuli and lowers the local nociceptive threshold (Barcikowska et al., 2020). Proinflammatory cytokines, including IL-1β, IL-6, and TNF-α, amplify prostaglandin synthesis and further excite nociceptors through neuroimmune interactions (Barcikowska et al., 2020). Oxidative stress, characterized by elevated malondialdehyde and reduced superoxide dismutase and glutathione peroxidase activity, impairs uterine perfusion through additional mechanisms (Barcikowska et al., 2020).

The prostaglandin pathway is self-contained and does not require an intravaginal trigger. Tampons do not cause primary dysmenorrhea. However, several mechanisms described below could plausibly amplify pain in individuals whose prostaglandin-driven nociceptive system is already primed during menstruation.

Central sensitization (the process by which repeated or intense peripheral nociceptive input lowers central pain thresholds) is increasingly recognized as a contributor to dysmenorrhea severity (Barcikowska et al., 2020). Women with primary dysmenorrhea exhibit lower pressure pain thresholds at remote body sites, consistent with a centrally sensitized pain state (Barcikowska et al., 2020). In the context of central sensitization, additional peripheral stimuli, including vaginal or cervical mechanical stimulation from a tampon, could be amplified beyond what would be experienced by a non-sensitized individual. A recent primary study confirmed that menstrual pain is associated with sexual pain specifically through visceral hypersensitivity, supporting a common neurobiological pathway through which intravaginal stimulation could interact with dysmenorrheic pain states (Reina et al., 2025).

### 5.2 Pelvic floor dysfunction and myofascial contributions

A case-control study (n = 84) found that women with primary dysmenorrhea had significantly higher prevalence of myofascial trigger points in the abdominal and pelvic floor muscles compared with pain-free controls (Serrano-Imedio et al., 2022). Pelvic floor hypertonicity (elevated resting tension in the levator ani and other pelvic floor muscles) commonly co-occurs with dysmenorrhea, endometriosis, and vulvodynia (Serrano-Imedio et al., 2022). Tampon insertion in the presence of pelvic floor hypertonicity may elicit a reflex guarding response or direct mechanical nociception, potentially contributing to a pain-cramping cycle. This mechanism is entirely speculative in the context of tampon use specifically, as no study has examined tampon use in women with pelvic floor dysfunction as the primary exposure, but the anatomy and neurophysiology support its plausibility.

### 5.3 Vaginal and cervical anatomy

The vaginal canal is lined by stratified squamous non-keratinized epithelium and is coated by a viscoelastic cervicovaginal fluid (CVF) that provides lubrication and maintains a protective mucus layer (Buchanan et al., 2023). The vaginal walls are highly vascularized, and this vascularity underlies the well-established property of vaginal tissue absorbing substances rapidly and without hepatic first-pass metabolism (Marroquin et al., 2024; Buchanan et al., 2023). The vaginal pH during the reproductive years is typically 3.8–4.5, maintained by Lactobacillus-produced lactic acid (Buchanan et al., 2023). These characteristics make the vaginal compartment highly responsive to both chemical and mechanical perturbations.

The cervix sits at the apex of the vaginal canal. A fully saturated or improperly seated tampon may contact or exert pressure against the ectocervix. The cervical os is richly innervated (Giovannetti et al., 2023), and cervical stimulation has documented pain-provoking effects, a principle leveraged, for example, in understanding intrauterine device (IUD) insertion pain (Arion et al., 2024). Whether typical tampon seating produces clinically relevant cervical contact pressure in the general population is unknown; this biomechanical interaction has not been characterized in the primary literature.

### 5.4 Mucosal desiccation and epithelial injury

One of the most directly documented mechanisms by which tampons interact with vaginal tissue is moisture extraction. Several older primary studies using colposcopy documented that tampon use, particularly superabsorbent tampon use, produces colposcopically visible alterations in the vaginal mucosa including epithelial drying, layering of cells, and microulceration (Barrett et al., 1977; Friedrich and Siegesmund, 1980; Weissberg and Dodson, 1983). These microulcerations were described as typically asymptomatic and self-resolving. However, a case series documented clinically apparent recurrent vaginal and cervical ulceration directly attributed to tampon use in a small number of patients; all cases resolved after tampon discontinuation (Weissberg and Dodson, 1983). In a comparative primary study, vaginal mucosal ulceration was statistically more common in users of rayon-polyacrylate superabsorbent tampons compared to other types, with microulcers hypothesized to arise from asymptomatic precursors (Friedrich and Siegesmund, 1980).

Epithelial microulceration represents a state of compromised mucosal barrier integrity. Under the conditions of dysmenorrhea, when prostaglandins and cytokines have already elevated local inflammatory tone, even minor mucosal disruption could activate or sensitize subepithelial afferent nerve terminals to produce or amplify discomfort. This remains a speculative but mechanistically coherent pathway.

The 2023 tampon safety assessment by Hochwalt and colleagues, using contemporary colposcopy across four commercial tampon products, found that tampon pressure points, rough edges, and sharp contact points were absent under study conditions, and the trial reported few complaints of burning, stinging, or irritation (Hochwalt et al., 2023). It should be noted that all authors of this study were employed by The Procter & Gamble Company at the time of publication. This finding suggests that modern well-designed tampons may have reduced, though not eliminated, the mucosal desiccation risk identified in earlier superabsorbent products. The products tested in this study were all from a single manufacturer (Tampax/Procter & Gamble), limiting generalizability.

### 5.5 Chemical irritation as a plausible pathway

Tampon materials contain detectable residues of metals, phthalates, and volatile organic compounds (characterized in Sections 6.3–6.5), which raise the mechanistic question addressed below: whether local chemical exposure at the vaginal mucosa contributes to irritation or nociceptor sensitization. From a mechanistic perspective, chemical irritants within the vaginal environment can trigger local inflammatory responses and, through prostaglandin-mediated pathways, sensitize nociceptors. If tampon materials leach irritating compounds into vaginal fluid, those compounds would be absorbed directly into the highly vascularized vaginal mucosa without hepatic first-pass metabolism (Marroquin et al., 2024).

A prospective cohort study of 259 healthy women (BioCycle study) found suggestive, though non-statistically significant, associations between tampon use and elevated urinary isoprostane (a validated biomarker of oxidative stress) compared with non-tampon use (Singh et al., 2019). The same study found non-significant trends toward higher mercury levels in tampon users (Singh et al., 2019). While neither finding reached statistical significance in this sample, the authors argued that tampons represent a potentially underappreciated source of chemical exposure warranting further study (Singh et al., 2019). This isolated finding should be weighed against other biomonitoring data: a 2024 systematic review of chemical exposure from menstrual products found no consistent evidence of elevated systemic biomarkers attributable to tampon use specifically (Marroquin et al., 2024), and tampon metal content analyses have generally reported concentrations within background dietary exposure ranges rather than a tampon-specific elevation (Shearston et al., 2024). In the context of dysmenorrhea, where oxidative stress is a recognized contributor to uterine ischemia and pain (Barcikowska et al., 2020), even modest tampon-attributable increases in systemic oxidative stress could theoretically interact with menstrual pain severity. This pathway is speculative and requires direct investigation.

### 5.6 Microbiome disruption and pro-inflammatory states

The vaginal microbiome during menstruation undergoes well-characterized shifts: menstrual blood raises vaginal pH, *Lactobacillus* relative abundance decreases, and microbial diversity temporarily increases (Hickey et al., 2013; Krog et al., 2022). Whether tampons exacerbate or attenuate this dysbiotic shift is not definitively established. A longitudinal study examining vaginal microbiome dynamics during tampon use found highly individualized community changes; in three of seven participants, diversity during pad use differed significantly from tampon-use cycles, suggesting product-specific effects (Hickey et al., 2013). The 2023 Hochwalt safety assessment found no adverse microbiome effects after one or more menstrual cycles with tampon use, though this was based on products from a single manufacturer and enrolled participants likely selected for absence of dysmenorrhea (Hochwalt et al., 2023).

Bacterial vaginosis (BV), a state of *Lactobacillus* depletion associated with anaerobic overgrowth, is a pro-inflammatory condition linked to elevated vaginal cytokines including IL-1β and IL-8. If tampon use were to promote BV in susceptible individuals, this would represent a pathway to elevated vaginal inflammatory tone during menstruation, potentially sensitizing local nociceptors. Conversely, a nested RCT in Kenya found lower rates of BV among menstrual cup users compared with pad users, with the proposed mechanism being that inert silicone material preserves vaginal pH more effectively than absorbent products (Van Eijk et al., 2019; Mehta et al., 2023). Whether cotton *versus* rayon tampons differ in their effects on BV rates has not been studied.

### 5.7 Summary of plausible mechanisms

The mechanisms reviewed above are summarized in Table 1. None is established in the context of tampon use and menstrual pain. All are mechanistically plausible based on adjacent evidence. The appropriate interpretation is that these mechanisms generate testable hypotheses.

**TABLE 1:** Plausible mechanisms linking tampon properties to menstrual discomfort.

Mechanism	Supporting evidence domain	Evidence strength
Mucosal desiccation from superabsorbent fibers causing epithelial microulceration and nociceptor exposure	Colposcopy studies (Barrett et al., 1977; Friedrich and Siegesmund, 1980; Weissberg and Dodson, 1983); (Hochwalt et al., 2023)	Moderate (mechanism demonstrated; pain linkage not studied)
Chemical leachates triggering local inflammation and prostaglandin sensitization	(Shearston et al., 2024); (Marroquin et al., 2024); (Singh et al., 2019)	Weak-to-moderate (chemicals present; leaching and vaginal inflammation not quantified)
Radial expansion and cervical contact pressure activating mechanoreceptors and cervical nociceptors	Tampon expansion geometry; cervical innervation literature	Speculative (no primary pressure mapping data)
Microbiome disruption increasing pro-inflammatory microbial activity during menses	Tampon microbiome studies (Hickey et al., 2013; Krog et al., 2022); BV as inflammatory state	Preliminary (effect on dysmenorrhea pathway not studied)
Pelvic floor muscle guarding in response to insertion discomfort in sensitized individuals	Pelvic floor dysfunction overlap with PD (Serrano-Imedio et al., 2022); visceral hypersensitivity (Reina et al., 2025)	Speculative-to-plausible
Central sensitization amplifying peripheral vaginal/cervical stimulation from tampon	Central sensitization in primary dysmenorrhea (Barcikowska et al., 2020); visceral hypersensitivity (Reina et al., 2025)	Indirect/plausible

## 6 Tampon materials, chemicals, and biocompatibility

### 6.1 Material composition

The absorbent core of commercially available tampons is composed of cotton (Gossypium hirsutum), rayon (viscose), or a cotton-rayon blend. Rayon is a regenerated cellulosic fiber produced from wood pulp through the viscose process, which involves treatment with carbon disulfide and sodium hydroxide. Cotton is a natural cellulosic seed fiber with a naturally waxed cuticle, crimp structure, and lower mean fiber surface roughness than rayon. Some products incorporate superabsorbent polymers such as carboxymethylcellulose. External covers are typically polypropylene nonwoven fabric. Applicators, where present, are made of either plastic (polyethylene, polypropylene) or cardboard.

### 6.2 Cotton *versus* rayon: comparative properties

Cotton and rayon differ in several properties with potential relevance to vaginal safety and comfort. Rayon fibers are generally smoother than cotton, but the primary driver of rayon’s absorbency advantage is fiber cross-sectional geometry rather than aspect ratio alone: trilobal-cross-section viscose fibers exhibit higher absorbency than round fibers of similar chemical composition and comparable mechanical characteristics, attributable to the greater specific surface area and bulkier fiber network that trilobal geometry produces (Harter et al., 2024). This geometric mechanism, not superabsorbent chemistry, is why trilobal fibers became the standard choice for high-absorbency tampon products after the 1980s toxic shock syndrome epidemic prompted manufacturers to move away from superabsorbent materials (Section 8.2). Cotton retains absorbed fluid more effectively under external compression. Both fibers are hydrophilic, but rayon absorbs fluid more rapidly.

The most clinically relevant comparative data on cotton *versus* rayon tampons comes from TSS research. An experimental study comparing the capacity of different tampon materials to amplify *S.taphylococcus aureus* TSST-1 production found substantially greater TSST-1 generation with non-cotton tampons, while all-cotton tampons produced no detectable TSST-1 under the same test conditions (Tierno and Hanna, 1994). The proposed mechanism was that rayon and superabsorbent materials alter vaginal oxygen concentration and fluid chemistry in ways that promote *Staphylococcus aureus* virulence gene expression (Tierno and Hanna, 1994; Vostral, 2017). Whether comparable material-specific differences exist for mucosal irritation, microbiome perturbation, or pain-relevant outcomes has not been studied.

The production of rayon through the viscose process has historically introduced chemical residue concerns. Analysis of rayon-containing tampons has detected carbon disulfide (a reproductive toxin used in rayon processing) in rayon-containing products but not in all-cotton products (Marroquin et al., 2024). This finding has not been replicated in peer-reviewed form as a primary study, but is noted in the systematic review by (Marroquin et al., 2024).

### 6.3 Metals

A 2024 analytical study by Shearston and colleagues measured metal and metalloid concentrations in 30 tampons representing 14 brands and 18 product lines. All 16 tested metals were detected in at least one sample, including arsenic, barium, cadmium, cobalt, chromium, copper, iron, manganese, mercury, nickel, lead, selenium, strontium, vanadium, and zinc. Lead was detected in all samples, with a geometric mean concentration of approximately 120 ng/g, while arsenic and cadmium were detected in most samples. The authors also reported higher lead concentrations in non-organic tampons and higher arsenic concentrations in organic tampons, indicating that organic labelling does not necessarily correspond to a lower overall contaminant burden. The authors of this study noted that trace metal presence in cotton-based products primarily reflects agricultural uptake, cotton plants absorb metals from soil affected by atmospheric deposition, wastewater application, and metal-containing pesticides and fertilizers, rather than deliberate inclusion during manufacturing (Shearston et al., 2024). However, this study measured metal content in tampon material rather than metal release during use or systemic absorption. A subsequent toxicological risk assessment addressed this gap directly: Jin and colleagues, publishing through the FDA’s Center for Devices and Radiological Health, measured both total metal content and metal release under exaggerated extraction conditions (50 °C, 24 h, acidified water) in 11 legally marketed US tampon products, then calculated margins of safety per ISO 10993 and FDA guidance (Jin et al., 2026). Nineteen metals were detected, including the ICH Class I metals arsenic, cadmium, and lead, but released amounts were a small fraction of total content (for example, lead: 237.2 ng per g total content vs. 3.5 ng per g released under exaggerated extraction), and the calculated margins of safety indicated negligible toxicological concern for all detected metals. This substantially narrows, though does not fully close, the gap identified above: the open question is no longer whether metals are present or release-capable in principle, but whether trace-level, safety-margin-cleared exposure has any bearing on local mucosal irritation specifically, a question distinct from systemic toxicity.

Following this publication, the FDA commissioned an independent systematic literature review and stated that, despite limitations in the available evidence, the findings did not identify safety concerns associated with tampon use and contaminant exposure. The FDA also emphasized that existing studies do not determine how much, if any, of the detected contaminants are released from tampons or absorbed through vaginal tissue. Similarly, the American College of Medical Toxicology issued a position statement concluding that current evidence does not support claims that tampons cause metal poisoning. Overall, the major unresolved limitation is that metal bioavailability from tampon materials under physiologic conditions has not yet been quantified, limiting the ability to infer clinical risk from measured material concentrations alone.

### 6.4 Volatile organic compounds and plastic additives

A systematic review identified phthalates, volatile organic compounds (VOCs), parabens, environmental phenols, fragrance chemicals, and dioxins and dioxin-like compounds in commercially available menstrual products (Marroquin et al., 2024). Among VOCs, carbon disulfide has been detected in rayon-containing products (Marroquin et al., 2024). A 2025 Environmental Science & Technology study examining plastic additives in single-use and reusable menstrual products detected phthalates (PAEs), organophosphate esters (OPEs), and alternative plasticizers in tampons, pads, and other products, with concentrations in tampons lower than in pads but not negligible (Cioni et al., 2025). An extractables and leachables investigation published in 2025 further characterized chemicals capable of migrating from menstrual product materials under simulated-use conditions (Marcelis et al., 2025).

Phthalates are endocrine-disrupting compounds used as plasticizers in manufacturing processes and potentially introduced through plastic packaging components or fragrance formulations. Epidemiological evidence associates phthalate exposure with adverse reproductive and menstrual health outcomes, including endometriosis and uterine fibroids, two conditions already characterized by severe dysmenorrhea (Marroquin et al., 2024). Whether phthalate leaching from tampons contributes meaningfully to circulating phthalate levels in users is not yet established.

### 6.5 Dioxins, furans, and bleaching residues

Dioxins and polychlorinated dibenzofurans are byproducts of the chlorine-based bleaching processes historically used to produce white tampon cotton and rayon. Modern regulations have substantially reduced but not eliminated dioxin concentrations in finished products through the adoption of elemental chlorine-free or totally chlorine-free bleaching. The FDA systematic literature review noted detectable polychlorinated dioxin concentrations in some tampon samples, at levels the FDA concluded did not represent a safety concern based on exposure estimates (U.S. Food and Drug Administration, 2024). The epidemiological review by Upson and colleagues identified discrepant conclusions across studies measuring chemicals including dioxins in menstrual products (Upson et al., 2022).

### 6.6 Vaginal mucosal permeability and chemical absorption

The vagina’s absorptive properties are relevant to any assessment of tampon chemical exposure. The vaginal and vulvar mucosa is highly vascularized and more permeable than comparable surface areas of keratinized skin on other body surfaces. Substances absorbed vaginally enter the systemic circulation without hepatic first-pass metabolism, a property exploited in vaginal drug delivery systems (Marroquin et al., 2024; Gerton and Mann, 2021). From menarche to menopause, the average individual who uses tampons will use in excess of 11,000 tampons across their lifetime, representing cumulative intravaginal contact with absorptive materials over thousands of hours (Marroquin et al., 2024). The pharmacokinetic implications of this cumulative exposure have not been quantified for the metal, phthalate, and dioxin residues documented in commercial tampons (Sections 6.3–6.5).

The anatomical sites at which these material and chemical interactions are most mechanistically relevant are illustrated in Figure 2. Panel A depicts tampon placement relative to the vaginal canal, cervix, and uterus; Panel B details three interaction zones–cervical contact at the tampon tip (Zone 1), the fiber-to-epithelial interface along the vaginal sidewall (Zone 2), and the vaginal microenvironment and pH (Zone 3) – each of which corresponds to a distinct mechanistic pathway discussed in this review.

**FIGURE 2:**
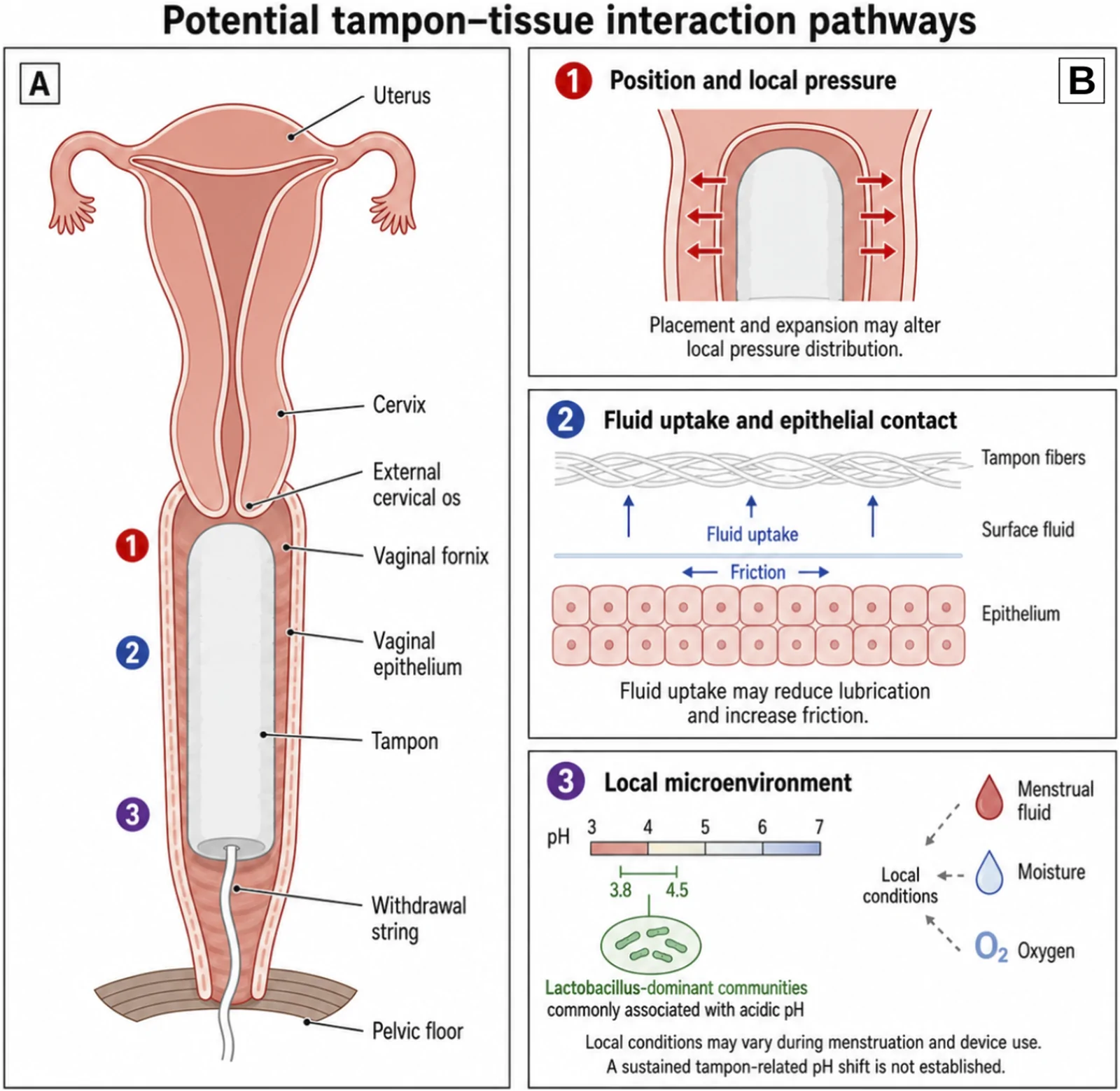
Vaginal Anatomy and Tampon-Tissue Interaction Zones. Schematic sagittal cross-section of the female reproductive tract with tampon *in situ* (Panel **(A)**) and three zoomed interaction zones (Panel **(B)**): cervical contact pressure (1), fiber-to-epithelial moisture wicking (2), and vaginal pH and microbiome disruption (3). Figure created by the authors using Canva (Canva Pty Ltd, Sydney, Australia).

### 6.7 Current safety conclusions and their limitations

The FDA’s 2024 regulatory review stated that the available evidence did not identify safety concerns associated with tampon use and contaminant exposure (U.S. Food and Drug Administration, 2024). This regulatory conclusion is important but must be interpreted in light of unresolved evidence gaps, including the limited data on long-term cumulative exposure and a lack of subgroup-specific studies in populations with endometriosis, fibroids, or other inflammatory gynecologic conditions. A subsequent toxicological risk assessment (Section 6.3) has since substantially narrowed, though not eliminated, the metal bioavailability gap specifically.

## 7 Absorbency, geometry, and mechanical design

### 7.1 Tampon geometry and expansion

In their dry, compressed state, tampons typically measure approximately 10–14 mm in diameter and 40–60 mm in length. Upon fluid absorption, tampon diameter may expand to approximately 20–28 mm at the widest point, representing a roughly 2- to 3-fold radial increase [patent literature; note: quantitative expansion data comes primarily from patent filings and proprietary technical specifications rather than peer-reviewed biomechanics studies]. This expansion occurs by absorptive swelling of the pledget matrix and is constrained by the pledget wrap. Non-destructive computed tomography imaging has directly characterized this internal structure: commercially available tampons exhibit an engineered density gradient, with lower density at the periphery and higher density at the core, a profile associated with better leakage performance than a uniform-density prototype tested in a consumer study (Hou and Acton, 2016). More recent micro-CT and pore-partition analysis of trilobal-fiber tampon proxies found 26 to 32 percent higher pore equivalent diameter, 41 percent greater expanded volume, and 18.7 percent higher absorbency compared to round-fiber counterparts, along with a systematic increase in pore size from the top to the bottom of the tampon, a gradient the authors attributed to one-sided liquid application and compression effects during manufacturing (Harter et al., 2026). These findings confirm that internal density and pore-structure gradients are an established, if underpublicized, feature of current tampon engineering.

Expansion patterns differ by material and product design: cotton-dominant pledgets tend to expand more uniformly and retain fluid under compression, while rayon-dominant pledgets expand faster and may have more asymmetric expansion geometries depending on fiber orientation. These differences in expansion dynamics directly determine the contact pressure exerted against vaginal walls and, in fully saturated or misplaced products, against the ectocervix. Commercial tampons are further distinguished by two patented expansion architectures: radial-expansion (“rolled-type”) designs, which expand primarily in diameter with minimal longitudinal change, and folded-expansion (“folded-type”) designs, which unfold both radially and longitudinally as pre-compressed folds absorb fluid (Glassman, 1991). Manufacturers have patented designs intended to maximize radial expansion specifically for comfort and fit, on the premise that expansion distributed along the tampon’s length rather than concentrated at one end improves anatomical fit (Agyapong, 2010), as well as folded pledget architectures intended to increase absorbent capacity and leakage protection (Jorgensen and Edgett, 2017). These remain patent-literature claims rather than independently validated comparative findings.

While the studies above characterize internal density and pore-structure gradients *ex vivo*, there are still no peer-reviewed primary studies characterizing tampon expansion using *in situ* measurement techniques, contact pressure mapping, or finite element modeling in vaginal phantom models. Whether these documented structural gradients translate into any measurable difference in vaginal contact pressure or comfort has not been studied; as the authors of the most recent micro-CT investigation themselves note, there remains a lack of engineering-focused research on their design and functionality (Harter et al., 2026). This represents a fundamental, and now more precisely defined, gap in the engineering characterization of the device.

### 7.2 Absorbency standardization

Tampon absorbency is standardized in the United States through the FDA-recognized ASTM F1230 Syngyna test method, which uses a standardized synthetic simulated menses fluid in a controlled apparatus. Absorbency tiers (junior/regular/super/super plus) are defined by Gram ranges of synthetic fluid absorbed. Critically, the Syngyna method measures fluid uptake but does not characterize expansion geometry, contact pressure, removal friction, or any biomechanical parameter relevant to user discomfort. The absorbency standard does not address pain-related design features, and no equivalent standard exists for comfort.

### 7.3 Friction during insertion, wear, and removal

Friction between tampon surface and vaginal mucosa is expected to occur at three clinically distinct moments, based on materials science principles rather than direct measurement: insertion (dry fiber contact with vaginal epithelium), mid-cycle wear (progressively saturated fiber in contact with mucosal surface), and removal (partially dry or partially saturated fiber dragged against epithelium). The most widely cited discomfort moment for tampons is removal of a partially dry (undersaturated) tampon, which is recognized in product guidance literature but has not been characterized in primary biomechanical studies.

From materials science principles, the friction coefficient at the tampon-tissue interface will depend on: tampon surface roughness (lower for rayon, higher for cotton); fiber orientation at the pledget surface; degree of fluid saturation (saturated fiber has lower friction coefficient than dry fiber); presence of coatings (none in current commercial products, to the authors' knowledge); and viscoelastic properties of the cervicovaginal fluid. No peer-reviewed study has measured the friction coefficient of commercial tampon materials against *ex vivo* vaginal tissue or validated phantom models under varying saturation states.

### 7.4 Pressure distribution and cervical contact

When a fully expanded tampon occupies the vaginal canal, it is expected to exert outward radial pressure against the vaginal walls, based on material expansion properties, and, depending on placement depth and expansion geometry, may exert apical pressure against the ectocervix. Neither has been directly measured *in situ*. This contact is implicitly acknowledged in the drug delivery literature, where tampon-like devices are described as delivering pharmaceutical agents to cervical tissue through distal fiber contact (Pollard, 2023). No primary study has characterized the magnitude or distribution of this pressure using pressure sensing arrays or computational modeling.

The clinical relevance of tampon-generated contact pressure depends on the mechanical properties and sensory threshold of the vaginal and cervical tissue at the time of use. During menstruation, elevated prostaglandins reduce the nociceptive threshold (Barcikowska et al., 2020), potentially lowering the pressure required to produce discomfort below what would be measurable in non-menstruating study participants.

### 7.5 Applicator design

Applicator-based tampons allow the pledget to be deposited at a predetermined depth without digital contact. Plastic applicators typically produce smoother insertion than cardboard applicators and allow more precise depth control. The design of the applicator plunger and tip geometry determines the force required for pledget deployment and the risk of incomplete deployment. The Health Canada safety review of the U by Kotex product documented cases where incomplete deployment or structural failure led to tampon fragmentation and retained product, triggering adverse reactions including abdominal pain (Health Canada, 2025). Applicator design, including insertion depth guides and ergonomic grip features, represents a tractable engineering design target.

## 8 Microbiome, infection risk, and toxic shock syndrome

### 8.1 The vaginal microbiome and menstrual products

A healthy reproductive-age vaginal microbiome is typically dominated by *Lactobacillus* species, particularly *Lactobacillus* crispatus and *Lactobacillus* iners, which produce lactic acid, hydrogen peroxide, and bacteriocins that maintain vaginal pH between 3.8 and 4.5 and provide colonization resistance against pathogenic bacteria (Krog et al., 2022). During menstruation, elevated vaginal pH from menstrual blood and cervical secretions disrupts this equilibrium: *Lactobacillus* relative abundance decreases, microbial diversity increases, and the community enters a transient dysbiotic state in many individuals (Krog et al., 2022).

A 2022 observational study found that vaginal dysbiosis was present in 40% of all samples across the menstrual cycle, rising to 58% of samples collected during the menstrual phase (Krog et al., 2022). Tampon use during this naturally dysbiotic period superimposes an absorbent, oxygen-modulating intravaginal device on an already-perturbed environment. Whether tampons accelerate, stabilize, or have neutral effects on menstrual phase dysbiosis has not been definitively established. The 2023 Hochwalt safety assessment, which included 16S rRNA gene sequencing analysis across four tampon products, reported no adverse effects on *Lactobacillus* presence or cell density after one or more menstrual cycles of tampon use (Hochwalt et al., 2023). These data are reassuring but derive from a single manufacturer’s products in a population selected for gynecological health.

Comparison with menstrual cups provides indirect evidence: a nested study within a Kenyan cluster RCT found lower BV prevalence in cup users *versus* pad users, with investigators hypothesizing that inert silicone preserves vaginal pH more effectively than absorbent materials (Van Eijk et al., 2019; Mehta et al., 2023). Whether this protective effect, if real, would translate to tampon-versus-cup comparisons is unknown, as absorbent tampons differ from non-absorbent silicone cups in ways beyond material alone.

### 8.2 Toxic shock syndrome: history, mechanism, and material-specific risk

Menstrual toxic shock syndrome (mTSS) is caused by vaginal colonization with TSST-1-producing *Staphylococcus aureus* in the context of superantigen-mediated immune dysregulation. The mTSS epidemic of 1980–1982 in the United States was directly linked to the introduction of highly absorbent tampons containing superabsorbent polyester and cross-linked carboxymethylcellulose fibers; withdrawal of these products and introduction of federal absorbency regulations substantially reduced mTSS incidence to approximately 0.5–1.0 per 100,000 users (Singh et al., 2019). Modern tampons instead achieve high absorbency primarily through fiber cross-sectional geometry rather than superabsorbent chemistry: trilobal viscose fibers, which have greater specific surface area than round fibers, produce comparable or superior absorbency without reintroducing superabsorbent materials, and manufacturers have specifically avoided superabsorbent chemistries because of their TSS association (Harter et al., 2024). Currently, approximately 30%–40% of healthy tampon users carry vaginal *S. aureus*, with approximately 5% of strains producing TSST-1, yet incidence of clinical mTSS remains rare (Vostral, 2017).

The material-specific dimension of TSS risk is well established: experimental studies comparing cotton and non-cotton tampons found substantially greater TSST-1 production in the presence of non-cotton (rayon and superabsorbent polymer) tampons, with all-cotton tampons producing no detectable TSST-1 under standard test conditions (Tierno and Hanna, 1994). The mechanism involves superabsorbent material altering vaginal fluid composition and oxygen tension in ways that upregulate *S. aureus* virulence genes (Tierno and Hanna, 1994; Vostral, 2017). A 2024 study identified the SaeRS two-component system as a key regulator of TSST-1 production and a potential antivirulence target (Dufresne et al., 2024), confirming the continued relevance of TSS as an active research area. These material-specific TSS differences, a well-established finding, indicate that tampon fiber composition has measurable biological effects on vaginal microbiology, establishing a precedent for material-specific effects relevant to other outcomes including mucosal irritation and inflammatory tone.

### 8.3 Menstrual products and reproductive tract infections

A 2025 systematic review evaluated the relationship between menstrual product use and reproductive and urogenital tract infections including HPV, HIV, BV, UTI, vaginitis, and candidiasis, searching PubMed, Web of Science, and the FDA MAUDE database through October 2024 (Krause et al., 2025). The review provided the most comprehensive synthesis to date of infection risk across menstrual product types, though it identified significant heterogeneity in study design and highlighted the need for standardized, prospective research to draw firm conclusions (Krause et al., 2025). In aggregate, available evidence does not identify tampons as a major driver of reproductive tract infections in otherwise healthy users, though specific conditions, including superabsorbent material use and extended wear duration, have established associations with elevated risk (Vostral, 2017; Krause et al., 2025).

## 9 Health equity and disparities in menstrual pain, diagnosis, and product access

### 9.1 Racial disparities in fibroid and endometriosis burden

Uterine fibroids affect approximately 80% of Black women by age 50, compared with approximately 70% of White women, and present earlier, larger, and more severely in Black women (Marcelis et al., 2025), with heavy menstrual bleeding from fibroids disproportionately burdening Black women across both commercial insurance and Medicaid populations (U.S. Food and Drug Administration, 2024). Endometriosis, which affects approximately 10% of reproductive-age individuals globally and is defined in part by severe dysmenorrhea, is diagnosed at substantially lower rates in Black women despite epidemiological evidence suggesting comparable or greater prevalence, with diagnostic delay exceeding 7–10 years on average and potentially twice as long for Black and Hispanic women (Marcelis et al., 2025). In one foundational study, approximately 20% of Black women undergoing laparoscopy for pelvic pain had endometriosis, yet 38% of these women had been pre-operatively misdiagnosed with pelvic inflammatory disease (Marcelis et al., 2025).

The implications for tampon research are direct: the populations with the highest burden of severe dysmenorrhea and pelvic pain, Black women and those with endometriosis or fibroids, are also the populations least represented in tampon safety and design research, which has been conducted predominantly in populations that are White, young, and free of major gynecological comorbidities.

### 9.2 Pain dismissal and normalization

A 2024 qualitative study of Black women with uterine fibroids and Latinas described a community norm of normalizing severe menstrual cramping as an expected feature of menstruation rather than a symptom warranting clinical attention, with participants reporting limited validation from healthcare providers and delayed care-seeking as a result (Orellana et al., 2024). A 2025 qualitative study similarly found that Black women with chronic pelvic pain experienced compounded racial and gender disparity in pain validation, with pain dismissed by clinicians and minimized by cultural expectations (Cush et al., 2025). These findings situate tampon discomfort within a broader structural context in which pain in Black women is systematically undertreated and underinvestigated.

### 9.3 Social determinants of dysmenorrhea

A systematic review of social determinants of health and dysmenorrhea found equivocal evidence linking socioeconomic status, geographic location, and race/ethnicity to dysmenorrhea outcomes, concluding that current evidence is insufficient to draw firm conclusions due to a lack of adequately powered large-scale studies rather than an absence of effect (Chen et al., 2024). This gap represents a clear priority for future research.

### 9.4 Differential product use and its implications for chemical exposure

As documented in Section 3.2, Black menstruators are more likely to use scented intimate care products and less likely to use unscented tampons or menstrual cups compared to White menstruators (Zota et al., 2023). Scented products introduce additional fragrance chemical exposure including phthalates and synthetic musks. Combined with greater fibroid and endometriosis burden (conditions potentially linked to endocrine-disrupting chemical exposure (Marroquin et al., 2024)), differential product use patterns may contribute to or interact with existing health disparities. This hypothesis requires rigorous investigation but points to the importance of designing tampon research that captures racial and socioeconomic subgroup variation rather than treating “tampon users” as a homogeneous population.

## 10 Canadian context: regulation, access, and research opportunity

### 10.1 Dysmenorrhea burden in Canada

The most comprehensive Canadian primary data on dysmenorrhea prevalence remains a survey published in 2005, which found that approximately 60% of Canadian menstruating women met criteria for primary dysmenorrhea, with 51% reporting activity limitation and 17% missing school or work due to pain (Burnett et al., 2005). No large-scale Canadian epidemiological update of this survey has been published to the authors' knowledge, representing a foundational gap in Canadian women’s health data. An updated Canadian prevalence study is overdue and would provide an important evidence base for health policy and research funding prioritization.

### 10.2 Period poverty and menstrual equity

A 2024 primary study found that approximately one in six (17%) Canadians who menstruate have experienced period poverty, defined as inability to access adequate menstrual products, education, or hygiene facilities, rising to one in four in households earning less than CAD 40,000 per year, with Indigenous, Black, and BIPOC communities, individuals with disabilities, people experiencing homelessness, immigrants, and gender-diverse individuals disproportionately affected (Health Canadaa). A parliamentary report by the Standing Committee on the Status of Women similarly found that 74% of Indigenous women and girls in remote communities reported difficulty accessing menstrual products “often” or “sometimes” (Standing Committee on the Status of Women FEWO, 2023). These access barriers affect dignity and participation and constrain product choice, potentially channeling lowest-income users toward the cheapest available products regardless of material composition or design quality.

A 2025 CMAJ commentary identified Canada as having made progress on legislative recognition of menstrual equity while continuing to face significant access gaps, particularly for northern and remote communities (Agyapong, 2010). Amendments to the Canada Occupational Health and Safety Regulations, in force since December 2023, now require federally regulated employers to provide free menstrual products to employees (Jorgensen and Edgett, 2017), meaningful policy progress that nonetheless does not address the design quality of the products provided.

### 10.3 Regulatory framework in Canada

In Canada, menstrual tampons are regulated under the Food and Drugs Act as Class II medical devices. Health Canada requires a medical device licence before market authorization, as well as compliance with quality manufacturing standards and post-market surveillance requirements. Labelling must include standardized absorbency information and warnings about TSS risk and symptoms (Health Canada, 2026). Health Canada has demonstrated active post-market surveillance capacity through its safety review of U by Kotex Sleek and Click tampons, which identified an increase in Canadian adverse event reports including tampon fragmentation and associated vaginal irritation, abdominal pain, and infection (Health Canada, 2025).

Canada’s regulatory framework does not currently include requirements for transparency in chemical composition disclosure, chemical extractables and leachables testing specific to the vaginal environment, or pain or comfort endpoints in premarket submissions. In the United States, the Menstrual Products Right to Know Act (introduced in Congress, 2024) would require manufacturers to disclose ingredients; no equivalent Canadian legislation exists at the time of writing, though the Standing Committee on the Status of Women has recommended increased transparency (Standing Committee on the Status of Women FEWO, 2023).

### 10.4 The Canadian research opportunity

Canada’s combination of a nationally funded healthcare system, multicultural and Indigenous population diversity, strong biomedical engineering capacity at institutions including the University of Calgary, and growing policy momentum on menstrual equity creates a favorable environment for research at the intersection of tampon design, women’s health, and health equity. No Canadian research program to the authors' knowledge has examined tampon materials, design, or biomechanics in relation to menstrual pain outcomes. This review represents a first step toward establishing the framework for such a program.

## 11 Biomedical engineering design opportunities

### 11.1 Material selection and biocompatibility framework

The sections above establish that meaningful engineering characterization already exists for several dimensions of tampon design, including internal density and pore-structure gradients (Section 7.1), fiber-geometry-driven absorbency (Section 6), and metal release evaluated under a formal toxicological risk assessment (Section 6.3), set against a documented history in which testing standards and regulatory oversight have not kept pace with product-level innovation (Section 1). What follows is not a proposed device or a set of validated interventions. It is a structured mapping of the gap between this existing engineering capability and what has actually been established, tested, or validated with respect to comfort or pain outcomes specifically. Each subsection below identifies a design dimension where relevant engineering knowledge already exists, states explicitly what is and is not known about its relationship to mucosal irritation or dysmenorrhea risk, and frames the resulting uncertainty as a concrete, testable research target rather than a design recommendation.

The primary material design opportunity for a lower-discomfort tampon lies in selecting fibers with superior biocompatibility profiles, not merely adequate safety, and optimizing surface properties to minimize mucosal irritation. ISO 10993 provides a framework for biocompatibility evaluation of medical devices, including cytotoxicity, sensitization, intracutaneous reactivity, systemic toxicity, and implantation testing (Kim et al., 2025). A 2022 primary study developed and validated a safety assessment scheme for menstrual cups composed of medical-grade silicone, providing a methodology directly adaptable to tampon material evaluation (Sica et al., 2022). It should be noted that all authors of this study were employed by The Procter & Gamble Company at the time of publication. This framework should be adapted explicitly for the vaginal environment, including mucosal irritation testing using vaginal epithelial cell models.

Vaginal-specific biocompatibility considerations include: pH response (materials should not significantly alter vaginal pH); mucin interaction (materials should not deplete CVF mucin layers); epithelial cell response (materials should not generate cytokine profiles consistent with inflammatory activation); and microbiome interaction (materials should not promote pathogenic microbial growth beyond the changes already attributable to menstruation itself) (Buchanan et al., 2023). None of these endpoints is currently required for tampon premarket clearance in Canada or the United States.

### 11.2 Fiber architecture and surface properties

This subsection is a proposed engineering approach rather than a validated finding; no published study has tested surface-roughness modification on a tampon product. Fiber surface roughness and orientation at the pledget surface directly determine the friction coefficient at the tampon-tissue interface (Section 7.3). Engineering strategies to reduce insertion and removal friction could include: fiber surface smoothing or controlled orientation; application of a biocompatible outer nonwoven layer with lower roughness than the interior pledget material; and fiber treatment or coating to maintain low friction coefficient across the saturation range from dry insertion to near-saturation removal. These modifications should be evaluated for their effect on absorbency capacity (a regulatory requirement) and on TSS risk parameters (a safety requirement).

### 11.3 Zonal absorbency and moisture management

A zonal absorbency architecture (in which the interior pledget maximizes fluid uptake while the outer layer retains moisture) could decouple the tampon’s absorbent function from its mucosal contact properties. This design principle would allow the core to remain highly absorbent while the outer surface maintains a hydrated, low-friction interface with the vaginal epithelium. Analogous moisture management principles are well-established in wound dressing design, where interfaces between highly absorbent cores and moisture-retaining outer layers are used to maintain tissue hydration while managing exudate.

Natural and synthetic hydrogel materials offer potential as outer layer components: hydrogels that absorb a limited amount of fluid rapidly create a gel interface layer that could reduce friction and maintain epithelial surface hydration (Kim et al., 2025). Materials such as sodium alginate, carrageenan, cellulose and hyaluronic acid have been evaluated in intravaginal drug delivery systems (Gerton and Mann, 2021; Kim et al., 2025; Osmałek et al., 2021) and indicate biocompatibility in vaginal environments. A 2021 primary biomaterials study suggests that hyaluronic acid-based mucoadhesive films remain in contact with vaginal tissue for extended periods and release compounds gradually (Gerton and Mann, 2021), establishing proof of concept for hyaluronic acid as a biocompatible intravaginal surface material. Such materials would require evaluation of their effect on menstrual fluid absorption kinetics (to ensure they do not impair the tampon’s core absorbency function) and on vaginal microbiome composition.

### 11.4 Lubrication strategies

The specific discomfort of dry tampon removal (a frequently reported user complaint) could be addressed through biocompatible surface lubrication. Candidates include: non-aqueous lubricating coatings that persist through the tampon’s wear duration; water-soluble hydrogel coatings that provide a gel layer during insertion and maintain lubrication at the surface; and mucoadhesive coatings that promote controlled fluid uptake to the outer layer. Mucoadhesive polymer systems evaluated for vaginal drug delivery, including hydroxypropyl methylcellulose (HPMC), poloxamer 407, and chitosan derivatives (Osmałek et al., 2021), represent a translatable precedent. The requirement for these coatings is that they must not impair absorbency, must be safe for vaginal use under ISO 10993 criteria, and must not alter the microbiome in ways that increase BV risk.

### 11.5 Controlled expansion geometry

Currently, tampon radial expansion on fluid absorption is governed primarily by material elasticity and pledget compression architecture, not by active geometric design. Engineering tampon expansion geometry to minimize contact pressure against the ectocervix while maintaining adequate vaginal wall apposition for fluid management is a tractable design problem. The following are proposed engineering strategies without prior experimental validation on a tampon device. Strategies could include: helical fiber winding that preferentially directs expansion radially rather than apically; zone-differentiated compression density (softer distal pledget, firmer proximal pledget) to reduce cervical contact force; and pleating or scoring of the pledget wrap to control expansion direction. Computational modeling, using finite element analysis of tampon expansion within a deformable vaginal phantom with material properties derived from published vaginal tissue biomechanics data, could provide design guidance prior to physical prototyping (Buchanan et al., 2023).

### 11.6 Chemical minimization

Several of these approaches already exist in niche commercial products; their inclusion here is a sourcing recommendation rather than a novel engineering proposal. A lower-discomfort tampon should also minimize chemical residue content as a design specification. Engineering and sourcing strategies include: use of certified organic, pesticide-free cotton; totally chlorine-free (TCF) bleaching rather than elemental chlorine-free or chlorine-containing processes; avoidance of fragrance additives; use of bioplastic or recycled material applicators that do not introduce phthalate plasticizers; and routine extractables and leachables testing as part of manufacturing quality assurance. The 2025 extractables and leachables investigation [39] provides a methodological template for establishing tampon-specific leachate profiles under simulated vaginal fluid conditions.

### 11.7 Evaluation and testing framework

A translational development pathway for a lower-discomfort tampon would require evaluation at multiple levels:

Bench testing: (i) absorbency testing using ASTM F1230 Syngyna method to confirm regulatory compliance; (ii) friction coefficient measurement of outer layer material against *ex vivo* porcine vaginal tissue or validated phantom material under varying saturation states; (iii) expansion geometry characterization using X-ray or MRI imaging in a validated phantom; (iv) contact pressure mapping using pressure-sensitive film arrays in a simulated vaginal model; (v) extractables and leachables profiling in simulated vaginal fluid.

*In vitro*/*ex vivo* testing: (i) cytotoxicity assay using vaginal epithelial cell (VEC) monoculture models (e.g., Ectocervical VK2/E6E7 cells); (ii) cytokine and inflammatory marker profiling from VEC models after 4–8 h material contact; (iii) vaginal microbiome interaction testing using *in vitro* models of vaginal microbial communities; (iv) ISO 10993 biocompatibility battery adapted for intravaginal contact (Buchanan et al., 2023).

Clinical evaluation: (i) crossover trial in healthy participants comparing prototype and commercial tampon comfort (VAS scores at insertion, wear, and removal); (ii) phase II crossover trial in participants with diagnosed primary dysmenorrhea, comparing tampon types on VAS menstrual pain, McGill Pain Questionnaire, and PROMIS pain interference scale across two menstrual cycles; (iii) vaginal microbiome assessment by 16S rRNA gene sequencing at baseline, mid-cycle, and post-menstrual time points.

## 12 Proposed design criteria for a lower-discomfort tampon

The following design criteria represent a synthesis of the mechanistic, material, and engineering evidence reviewed above. These criteria constitute research targets rather than a finalized device design. A full patent landscape review was not conducted as part of this work; however, relevant comfort- and expansion-oriented patents from major manufacturers are discussed in Section 7.1 (Glassman, 1991; Agyapong, 2010; Jorgensen and Edgett, 2017). Before any development program proceeds, a systematic and comprehensive review of the existing patent and commercial product landscape should be conducted, since several manufacturers already market comfort-related features (e.g., smooth-tip applicators) without public efficacy data. These criteria require bench, *in vitro*, and clinical testing before any claim of clinical benefit could be made. These proposed design criteria are summarized in Table 2.

**TABLE 2:** Proposed engineering design criteria for a lower-discomfort tampon.

Design parameter	Current state (commercial)	Proposed specification	Rationale
Outer surface friction coefficient	Dry compressed cotton or rayon fiber; friction increases upon partial saturation	Biocompatible low-friction outer layer (e.g., hydrogel or mucoadhesive coating); friction coefficient maintained below critical threshold across saturation range	Reduce epithelial microabrasion during insertion and removal; reduce removal discomfort from partially dry fiber
Mucosal hydration at contact surface	Fiber surface (cotton/rayon geometry, not superabsorbent chemistry) may still locally reduce moisture at the contact surface during high-absorbency phases	Outer layer retains or donates moisture to vaginal epithelium while inner core absorbs fluid	Prevent colposcopically documented epithelial drying and microulceration (Barrett et al., 1977; Friedrich and Siegesmund, 1980; Weissberg and Dodson, 1983)
Radial expansion geometry	Radial expansion based on material swelling properties; not actively designed	Controlled expansion pattern preferentially limiting apical (cervical-directed) expansion; computational model-validated	Reduce cervical contact pressure and cervical mechanoreceptor stimulation
Chemical residue profile	Metals, VOCs, phthalates detectable in current products (Shearston et al., 2024; Marroquin et al., 2024; Cioni et al., 2025)	Totally chlorine-free materials; no fragrance; organic, pesticide-free cotton; phthalate-free applicators; extractables and leachables profile established and minimized	Reduce potential for chemical-mediated local inflammation or endocrine disruption
Biocompatibility standard	FDA 510(k) general biocompatibility requirements	ISO 10993-adapted vaginal-specific battery including VEC cytotoxicity, cytokine profiling, and vaginal microbiome interaction testing	Establish mucosal safety beyond standard equivalence review
Absorbency	Tiers standardized by ASTM F1230 Syngyna method	Maintain or improve absorbency within regulatory tier specifications	Ensure comfort design does not compromise core function
Applicator design	Plastic or cardboard applicator; no depth guide	Applicator with depth guide; ergonomic, smooth deployment; biodegradable or bioplastic material	Reduce insertion-related trauma; reduce phthalate exposure from plastic applicator

The proposed design architecture is illustrated schematically in Figure 3, alongside the current standard commercial tampon. Critically, the proposed design is not a pre-hydrated or gel-saturated device; it retains a dry high-absorbency inner core and introduces a porous intake tip to preserve menstrual fluid uptake, with a thin lubricious coating applied to the sidewall surface only. This configuration is intended to reduce friction at the fiber-to-epithelial interface during insertion and removal without impairing absorption, altering expansion geometry, or introducing retained surface moisture that could affect the vaginal microenvironment. A proposed five-step translational validation pathway is depicted in the lower panel of Figure 3.

**FIGURE 3:**
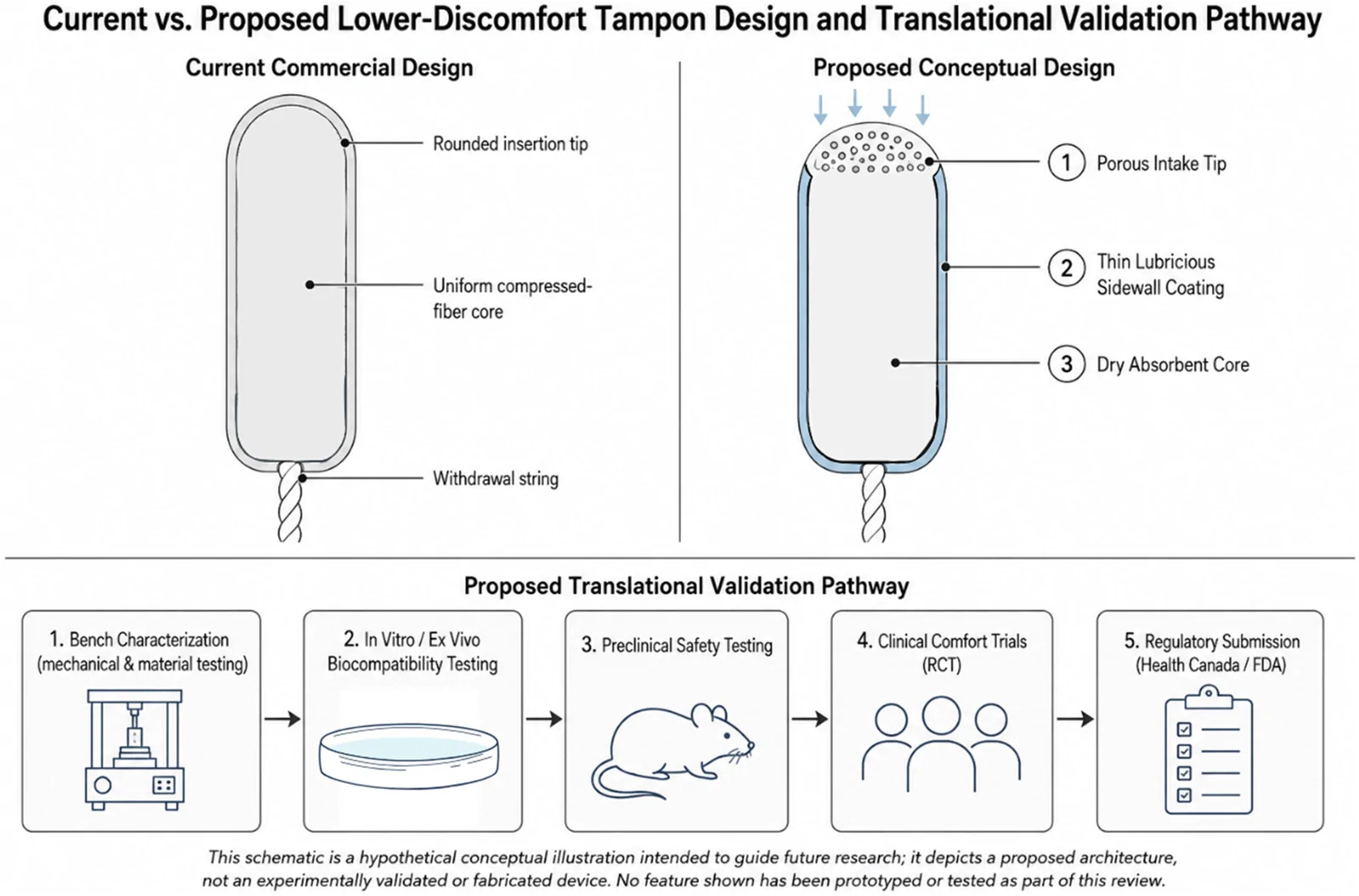
Current vs. Proposed Lower-Discomfort Tampon Design and Translational Validation Pathway. This schematic is a hypothetical conceptual illustration intended to guide future research; it depicts a proposed architecture, not an experimentally validated or fabricated device. The panel contrasts a standard commercial tampon with a conceptual design featuring a porous intake tip, thin lubricious sidewall coating, and dry absorbent core, alongside a proposed five-step validation pathway from bench characterization to regulatory submission. No feature shown has been prototyped or tested as part of this review. Figure created by the authors using Canva (Canva Pty Ltd, Sydney, Australia).

Schematic comparison of a standard commercial tampon against a proposed architecture featuring a porous intake tip, thin lubricious sidewall coating, and dry absorbent core, with a five-step validation pathway from bench characterization to regulatory submission.

## 13 Limitations and conflicting evidence

The mechanisms proposed in this review should be weighed against a body of evidence that is, in several cases, more reassuring than alarming for currently marketed products. This section consolidates that counter-evidence, which appears at points throughout the manuscript, so it can be evaluated as a whole rather than as isolated caveats.

### 13.1 Mucosal injury

The colposcopy studies most frequently cited for tampon-associated mucosal desiccation and microulceration were conducted between 1977 and 1983, using superabsorbent polyester and cross-linked carboxymethylcellulose fiber technologies that were withdrawn from the market following the 1980-1982 toxic shock syndrome epidemic and subsequent federal absorbency regulation. The most recent colposcopic assessment of commercial products, using four contemporary tampon lines, found no pressure points, rough edges, or sharp contact points, and few complaints of burning, stinging, or irritation. While this study’s generalizability is limited by single-manufacturer sponsorship, its findings meaningfully temper the applicability of the older mucosal injury literature to products currently on the market.

### 13.2 Chemical exposure

Following publication of metal(loid) content data in commercial tampons, both the FDA’s commissioned systematic literature review and a position statement from the American College of Medical Toxicology concluded that available evidence does not support a safety concern or a causal link between tampon use and metal poisoning. A separate cohort study measuring urinary oxidative stress and mercury biomarkers in tampon users found associations that did not reach statistical significance. The chemical-exposure mechanisms proposed in this review should be read against this backdrop: detectable material content has been established, but neither meaningful vaginal bioavailability nor a clinical health effect has been.

### 13.3 Microbiome disruption

The most recent 16S rRNA sequencing assessment of tampon use across one or more menstrual cycles found no adverse effect on *Lactobacillus* presence or vaginal community composition. This finding is more reassuring than the theoretical microbiome-disruption mechanism proposed in Section 5.6 would predict, though it is again limited to a single manufacturer’s products in a population selected for gynecological health.

### 13.4 Health equity and social determinants

A systematic review specifically examining social determinants of health and dysmenorrhea found the existing evidence base equivocal on associations between race, ethnicity, geography, and socioeconomic status and dysmenorrhea severity, concluding this reflects a lack of adequately powered studies rather than an absence of effect. This review’s equity framing (Section 9) should therefore be read as identifying a research gap and a plausible convergence of risk factors, not as an established causal pathway between differential product use and menstrual pain disparities.

### 13.5 Overall appraisal

Taken together, for nearly every mechanism proposed in this review, there is at least one contemporary study using current products or methods that did not find evidence of the harm being hypothesized. This does not invalidate the case for further investigation; the absence of adverse findings in small, often industry-funded, single-manufacturer studies is not equivalent to a well-powered independent safety evaluation across a diverse population, and this asymmetry is itself part of the evidence gap this review identifies. But it does mean the current weight of evidence, where it exists at all, leans toward reassurance for modern commercial products specifically, and this review’s proposed mechanisms should be understood as motivating hypotheses for that missing independent evaluation, not as findings in their own right.

## 14 Discussion: research gaps and future study designs

The following research gaps were identified through this review. They are listed in approximate order of foundational priority, from basic characterization to translational trials.

Gap 1: No biomechanical characterization of tampon-vaginal wall interaction during use. Contact pressure mapping, friction coefficient measurement, and expansion geometry characterization of commercial tampons using validated vaginal phantom models do not exist in the peer-reviewed literature (Buchanan et al., 2023). This is the most fundamental engineering data gap. Priority: establish a validated phantom model and perform comparative characterization across commercial product types.

Gap 2: No clinical trial comparing tampon types on menstrual pain outcomes. Building on the evidence gap established in Section 4.1, a crossover trial randomizing participants with primary dysmenorrhea to different tampon materials (cotton vs. rayon vs. organic cotton) across matched menstrual cycles, with validated pain outcomes (VAS, PROMIS Pain Interference) as primary endpoints, is the most direct way to close it.

Gap 3: No *in vitro* characterization of mucosal inflammatory response to tampon materials. Vaginal epithelial cell models are established and commercially available (Kim et al., 2025). A study comparing pro-inflammatory cytokine profiles (IL-6, IL-8, TNF-α, IL-1β) from VEC cultures after contact with cotton vs. rayon vs. blended tampon materials under simulated menstrual conditions would provide mechanistic data on material biocompatibility beyond current requirements.

Gap 4: Chemical bioavailability across vaginal mucosa is not quantified. Establishing leachate concentrations from tampon materials under simulated use conditions, combined with *ex vivo* vaginal permeation studies, would quantify the dose delivered to the vaginal epithelium and provide the missing input for risk assessment.

Gap 5: No stratified data on tampon discomfort in populations with dysmenorrhea-associated conditions. The hypothesis that tampons exacerbate pain in women with endometriosis, fibroids, pelvic floor dysfunction, or vulvodynia (by engaging pain pathways that are not activated in healthy menstruators) is untested. A prospective survey-based study recruiting women with these conditions could establish subgroup-specific adverse event profiles.

Gap 6: No updated Canadian dysmenorrhea prevalence data. The only national Canadian estimate is from 2005 (Burnett et al., 2005). An updated population-based study, stratified by age, geography, Indigenous identity, and socioeconomic status, is a public health priority.

Gap 7: Indigenous menstrual health research is absent from the literature. No published primary study examines tampon use, menstrual product choice, or dysmenorrhea outcomes in Canadian Indigenous communities. This gap reflects broader underrepresentation of Indigenous women in reproductive health research and should be addressed through community-partnered research design.

Gap 8: No human factors or user-centered design study for tampon design optimization. Patient-reported outcome measures (PROMs) tailored to intravaginal menstrual product comfort have not been developed or validated. A PROM development study using standard psychometric methods, with qualitative phases engaging diverse menstruator populations, would provide the outcome measurement infrastructure for future clinical trials.

## 15 Conclusion

Dysmenorrhea is a near-universal condition among menstruating individuals, carrying an enormous global and Canadian health, economic, and quality-of-life burden, yet tampons, among the most widely used intravaginal menstrual devices, have been designed without pain minimization as a formal design or regulatory endpoint. As established in Section 4.1, no peer-reviewed clinical trial directly links tampon use or materials to menstrual cramp severity. Multiple mechanistically plausible pathways may exist by which tampon material properties and biomechanical design contribute to or amplify pelvic discomfort in susceptible users, spanning mucosal desiccation, chemical irritation from residues of metals, phthalates, VOCs, and dioxin-related compounds of uncharacterized bioavailability, microbiome perturbation, and mechanical pressure. Whether these mechanisms actually operate, and whether they converge in the populations most burdened by dysmenorrhea, including Black and Indigenous menstruators, is an open empirical question rather than an established finding. What is established is that these populations are simultaneously the most underserved by current product design and research, which is reason enough to prioritize the investigation this review calls for. Canada offers a distinct opportunity to close this gap, with a policy environment increasingly oriented toward menstrual equity and a biomedical engineering infrastructure capable of moving from these identified mechanisms to testable design specifications and clinical evaluation.

Biomedical engineering provides the framework to move from documented evidence gaps to testable design specifications: material selection with vaginal-specific biocompatibility standards, friction-reducing surface architectures, controlled expansion geometry, chemical minimization, and a structured preclinical-to-clinical evaluation pathway. The proposed design criteria in this review are not claims of novelty and require patent landscape review before development. They represent a synthesis of the evidence into a tractable translational target.

As documented in Section 1, testing standards and regulatory oversight have not kept pace with product-level innovation, even where absorbency and safety engineering have continued to evolve. This does not establish that tampon design causes menstrual pain; it identifies a testable hypothesis, one with sufficient mechanistic plausibility and unmet clinical need to justify direct empirical investigation using the engineering and evaluation framework proposed here.
